# Wetland vegetation cover changes and its response to climate changes across Heilongjiang-Amur River Basin

**DOI:** 10.3389/fpls.2023.1169898

**Published:** 2023-08-04

**Authors:** Xinyue Chang, Lingxue Yu, Guangshuai Li, Xuan Li, Lun Bao

**Affiliations:** ^1^ Remote Sensing and Geographic Information Research Centre, Northeast Institute of Geography and Agroecology, Chinese Academy of Sciences, Changchun, China; ^2^ University of Chinese Academy of Sciences, Beijing, China; ^3^ College of Geography Science, Changchun Normal University, Changchun, China; ^4^ School of Geomatics and Prospecting Engineering, Jilin Jianzhu University, Changchun, China

**Keywords:** Heilongjiang-Amur River Basin, climate change, partial correlation, transboundary region, wetland vegetation cover

## Abstract

The Heilongjiang-Amur River Basin is one of the largest and most complex aquatic systems in Asia, comprising diverse wetland resources. The wetland vegetation in mid-high latitude areas has high natural value and is sensitive to climate changes. In this study, we investigated the wetland vegetation cover changes and associated responses to climate change in the Heilongjiang-Amur River Basin from 2000 to 2018 based on the growing season (May to September) climate and LAI data. Our results indicated that the wetland LAI increased at 0.014 m^2^·m^-2^/yr across Heilongjiang-Amur River Basin with the regional climate showed wetting and warming trends. On a regional scale, wetland vegetation in China and Russia had positive partial correlation with solar radiation and minimum air temperature, with precipitation showing a slight lag effect. In contrast, wetland vegetation in Mongolia had positive partial correlation with precipitation. These correlations were further investigated at different climate intervals. We found the precipitation is positively correlated with LAI in the warm regions while is negatively correlated with LAI in the wet regions, indicating an increase in precipitation is beneficial for the growth of wetland vegetation in heat sufficient areas, and when precipitation exceeds a certain threshold, it will hinder the growth of wetland vegetation. In the cold regions, we found solar radiation and minimum air temperature are positively correlated with LAI, suggesting SR and minimum air temperature instead of mean air temperature and maximum air temperature play more important roles in affecting the wetland vegetation growth in the heat limited areas. The LAI was found to be negatively correlated with maximum air temperature in the arid areas, indicating excessive temperature would inhibit the wetland vegetation growth when the water is limited. Our investigation can provide a scientific foundation for the trilateral region in wetland ecosystem protection and is beneficial for a more comprehensive understanding of the responses of wetlands in the middle and high latitudes to climate change.

## 1 Introduction

Wetlands provide almost one of third nature’s contribution to people (NCP) in the world, which benefits both humankind and nature, including carbon sequestration, flood control, water purification, and climate change mitigation (Millennium ecosystem assessment, 2005; Díaz et al., 2019). These valuable contributions make wetlands drive the changes in global water cycles, carbon sequestration, and the Earth’s climate. As such, preserving the ecosystem is crucial for maintaining the health and sustainability of our planet. The climate is the key driver that controls the growth and decline of wetlands, and its changing patterns due to climate change will have far-reaching effects on wetland ecosystems, from material and energy cycles to the diverse range of animals that depend on these habitats (Burkett and Kusler, 2000). Wetlands are highly responsive to climate change, with their patterns, components, distribution, and ecological effects closely intertwined with climate influences (Turner et al., 2000; Lahmer et al., 2001). Changes in rainfall intensity and frequency have significant impacts on wetlands, from direct and indirect effects to alter the biogeochemistry and function of the wetland (Salimi et al., 2021). Air temperature is expected to affect photosynthesis and act on vegetation growth (Kayranli et al., 2010; Peng et al., 2013; Gallego-Sala et al., 2018). Therefore, wetland vegetation change can significantly impact wetland ecosystems, accelerating biogeophysical land-atmosphere water and heat exchanges, with implications for the Earth system (Myneni et al., 1997; Yu et al., 2020; Yan et al., 2022).

A wide range of ecological processes is being impacted by the ongoing and expected acceleration of climate change in mid-high latitudes, as a result of these environmental changes, the productivity, and composition of vegetation have been directly affected (Goetz et al., 2005; French et al., 2008). Currently, research on how vegetation responds to climate change mostly focuses on forest and grassland vegetation, with very little attention paid to the influence of climate change on wetland vegetation (Wei et al., 2017; Chen et al., 2020; Ji and Fan, 2020). Over half of the world’s wetlands are located at latitudes above 50° in the northern hemisphere, against the backdrop of global warming, permafrost has degarded and vegetation in mid-high latitudes have experienced a greening trend over the past two decades (Menzel and Fabian, 1999; Lucht et al., 2002; Avis et al., 2011). Wetlands are a transitional system of aquatic and terrestrial ecosystems, with significant implications for energy and water cycles, including changes in surface albedo, heat, and moisture transport, so they are sensitive and vulnerable to climate change (IPCC, 2013; Peng et al., 2014; Yu et al., 2017; Masson-Delmotte et al., 2021).

The Heilongjiang-Amur Basin (HARB), is a cross-border area including China, Mongolia, and Russia, with different natural conditions. Therefore, understanding the differences in wetland vegetation responses to climate change in the HARB could help improve our understanding of cross-border wetland vegetation in mid-high latitudes. There have been significant improvements in the wetland phenology, and a predominance of vegetation turning green (Zhang et al., 2013). Because of the high vegetation coverage and abundant vegetation types in HARB, subsequent studies used to focus on the whole basin and prefer the normalized differential vegetation index (NDVI) datasets to illustrate the changes in vegetation activities to measure plant productivity (Chuai et al., 2013; Bao et al., 2014; Du et al., 2017; Chu et al., 2019; Lin et al., 2020). NDVI can be overestimations at the beginning and the end of the growing season (Islam and Bala, 2008). The mechanism of vegetation sustaining life on Earth is documented as the one-sided leaf area per unit of the ground surface area, which is the leaf area index (LAI) (Ross, 1981; Chen and Black, 1992; Smith, 2004). LAI can be a required variable for analyzing the background of growing season vegetation coverage and measuring both the process of vegetation growth and canopy reflectance. (Piao et al., 2003; Montandon and Small, 2008; Liu and Lei, 2015). The wetland vegetation phenological change is contingent upon two pivotal factors, namely aqueous levels and the carbon cycle, two crucial factors that shape the flourishing status of these invaluable ecosystems. As for phenological changes that were most pronounced in spring events than the other seasons, the growing season is a key indicator of wetland vegetation growth conditions and has been widely used in previous research (Menzel et al., 2006; De Jong et al., 2011). Nevertheless, there is an information deficiency regarding its spatial wetland vegetation response to climate change and analysis of the significant climate factors contributing to the HARB growing season wetland LAI variations.

This study aims to investigate the spatiotemporal changes in the wetland leaf area index (LAI) during the growing season from 2000 to 2018 in the HARB. By using partial correlation analysis, we aim to quantify the correlation of precipitation, solar radiation (SR), mean air temperature (T_avg_), maximum air temperature (T_max_), and minimum air temperature (T_min_) to the wetland LAI in the three countries under different natural and cultural conditions in HARB. In addition, we will discuss the annual climate change background and the unique relationship between LAI and climate drivers. Our findings are expected to provide valuable insights into the sustainable development of wetland ecosystems and enhance the understanding of climate factors’ effects on wetland vegetation. This research will also contribute to the understanding of the response of cold region ecosystems to global climate change.

## 2 Study area and datasets

### 2.1 Study area

**Figure 1:**
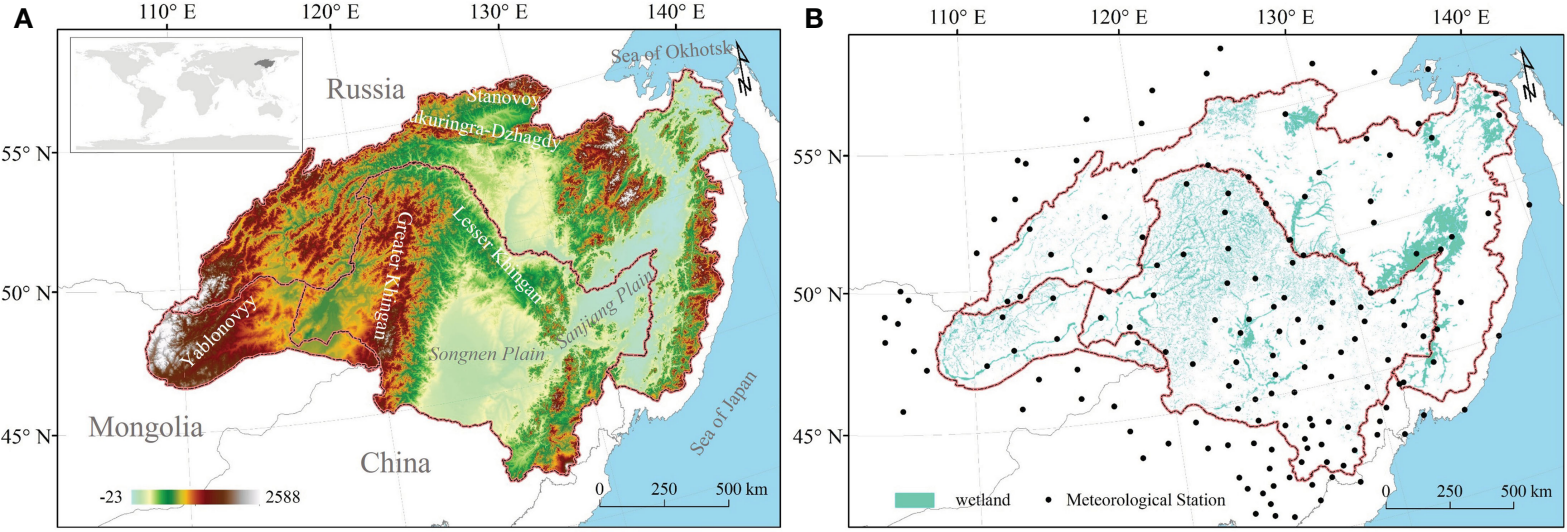
Geographical location and meteorological stations of HARB **(A)** Location map of the HARB showing digital elevation (DEM). **(B)** The spatial distribution of HARB wetlands and meteorological stations.

**Figure 2:**
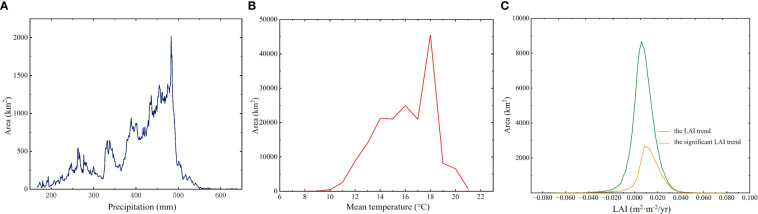
The frequency distribution of growing season precipitation value **(A)**, temperature value **(B)**, and trend of LAI **(C)** across HARB.

HARB, situated in the northeastern part of the Asian continent within the middle and high latitudes, includes expansive and fertile wetlands (Pervushina, 2012). Encompassing approximately 2,080,776 km^2^ of land, spanning from 107°31′ to 141°14′E in longitude and from 41°42′ to 55°56′ in latitude (**Figure 1**). The western and eastern portions of this basin have quite different climates. The HARB is influenced by the cold and dry monsoon from Siberia during winter, and the wet and humid monsoon from the Sea of Okhotsk and the Sea of Japan during summer, during the growing season, Okhotsk and Japan Sea are the main dominant reason for warm and humid monsoons (Chu et al., 2019). HARB wetland experienced a wide range of precipitation and air temperature levels from east to west, and higher rainfall and air temperature occurred in the growing season. Mean growing season precipitation increased with longitude, ranging from 166 - 645 mm (**Figure 2A**). The distribution of strong precipitation is mostly concentrated along the Amur River, Greater and Lesser Khingan Mountains and Stanovoy. Mean growing season air temperature decreased in higher elevations and latitudes, ranging from 6 – 21°C (**Figure 2B**).

The HARB is characterized by a diversity of vegetation types. Chinese territory contained agricultural regions in plains and sizable sections of wild vegetation in mountains, the Mongolian part of HARB mainly contains pastureland and the Russian part covers natural vegetation. Northeast China (42%), the Russian Far East Area (50%), and the Northeast of Mongolia (8%) comprised by the HARB, with the eleventh largest wetland in the world and covers 8% of the wetland area (Chu et al., 2019). Mixed forest occupied most of the Greater and Lesser Khingan Mountains in China, while vast stretches of larch left sparsely forested swamps in the Russian region, and almost all grassland is located in the Mongolian region (Egidarev et al., 2016a; Chu et al., 2019). Several mountain ranges run across HARB from north to south. Where mountains like Yablonovyy and Greater Khingan, stretch forth in a southerly direction towards the north. In the west-to-east direction, Stanovoy and Tukuringra-Dzhagdy stand firm at the northern border of HARB. Songnen and Sanjiang plains are in the south and east respectively. The varied topography coupled with distinct socio-economic objectives and cultural practices of China, Russia, and Mongolia has rendered the region a complex mosaic relationship between wetlands and climate change.

### 2.2 Data

The moderate Resolution Imaging Spectrometer (MODIS) LAI data were obtained for this research from 2000 to 2018 to examine vegetation growth and its response to climate change. We adopted LAI (MOD15A2H) data from 2000 to 2018, with a temporal resolution of 8-day and 500 m spatial resolution, and resampled to 1km by the resample tool in ArcGIS 10.2. In this paper, the solar radiation was extracted from the monthly value dataset TerraClimate, with a spatial resolution of a 0.5° and resampled to 1km (Abatzoglou et al., 2018). Both LAI and solar radiation datasets were acquired on the Google Earth Engine (GEE) platform. As it is the season when vegetation grows most prolifically, May to September is adopted to obtain a deeper comprehension of the vegetation growing cycle (Peng et al., 2011; Piao et al., 2011; Liu and Lei, 2015; Robinson et al., 2018; Wang et al., 2020; Yang et al., 2020). Precipitation and air temperature data were from 163 stations, 103 from the total observation sites containing valid data and 60 nearby sites are supplementary data in addition. The climate data adopted in this work are monthly precipitation, solar radiation (SR), mean (T_avg_), maximum (T_max_), and minimum (T_min_) air temperatures from 2000 to 2018. We calculate the monthly precipitation, T_avg_, T_max_, and T_min_ from daily data selected between May and September to obtain the growing season precipitation and air temperature. We used the ANUSPLIN software to interpolate all observation data, including precipitation and temperature, with the thin plate spline method, using DEM as a covariate (Hijmans et al., 2005). To match the resolution of the wetland vegetation data, we set the spatial resolution to 1 km. As the stations belong to 3 countries, the data have different sources: 65 were from China Meteorological Administration (CMA) (http://data.cma.cn), 6 stations in Mongolia were collected from the Mongolian Academy of Sciences; 32 Russia observation sites were from National Center for Environmental Information (https://www.ncei.noaa.gov/maps-and-geospatial-products). The wetland map was derived from LULC data with a spatial resolution of 1km based on a human-computer interaction interpretation, with a total accuracy of 90.800%, which was verified by comparing the classification results with higher resolution Google earth images (Liu et al., 2022). To analyze the response of growing season wetland vegetation LAI to climate change, this study assumed that the wetland area remained unchanged in 2015.

## 3 Methods

### 3.1 Trend analysis method

Wetlands are highly sensitive to climate change, and as a result, variations in climate can directly impact the structure and physiology of vegetation. Based on the LAI time series in the HARB in the growing season during 2000-2018, linear regression models due to ordinary least squares (OLS) were adopted to calculate the spatial and temporal patterns of wetland vegetation dynamics (Jiang et al., 2017; Meng et al., 2019). The student-t method was also used to determine whether the parameter trend was significant at both the basin and pixel scales. Most notably, the student-t results only indicate the confidence level of the parameter’s trend and not its speed (Chen et al., 2011; Yang et al., 2020). The slope of the linear regression was calculated for each grid to provide a more precise understanding of the associated trend.


(1) $$\theta_{slope}=\;\frac{\left(n\;\times \;\sum_{i=1}^{n}i\;\times LAI_{i}\right)\;-\left(\sum_{i=1}^{n}i\;\sum_{i=1}^{n}LAI\right)}{n\;\times \;\sum_{i=1}^{n}i^{2}-\;{\left(\sum_{i=1}^{n}i\right)}^{2}}$$

Where $\theta_{slope}$ represents the interannual variation slope of a pixel LAI; n is the number of years analyzed, i.e. 19 years for this study; $LAI_{i}$ represents the LAI value of the year i ; a positive value of slope indicates that the LAI illustrates an increasing trend and vice versa.

### 3.2 Correlation analysis

In light of the high correlation in the climate factors, we studied the correlation coefficients to determine the relationship between LAI and precipitation, SR, mean, maximum, and minimum air temperature, or during the growing season at pixel scales in the HARB (Peng et al., 2013; Chang et al., 2014). The purpose of this study was to gain a better understanding of the specific climate factors that have an impact on wetland vegetation.


(2) $$R_{xy}=\;\frac{\sum_{i=1}^{n}\left(x_{i}-\;\bar{x}\right)\left(y_{i}-\;\bar{y}\right)}{\sqrt{\sum_{i=1}^{n}{\left(x_{i}-\;\bar{x}\right)}^{2}}\sqrt{\sum_{i=1}^{n}{\left(y_{i}-\;y\right)}^{2}}}$$

Where $\;R_{xy}\;$ is the correlation coefficient between variable x and y ; $x_{i}$ is the growing season LAI for year i , and $y_{i}$ is the growing season mean air temperature or total precipitation for year i ; x and y represent long-term (2000 - 2018) averaged growing season LAI and different climatic drivers.

To assess the dominant factors from different climate variables, the partial correlation could measure the strength of a linear relationship between wetland vegetation and these climate factors. Meanwhile, partial correlation considers the influence of other variables (Zhang et al., 2011).


(3) $$R_{xy_{1}y_{2}}=\;\frac{R_{xy_{1}}-\;R_{xy_{2}}R_{y_{2}y_{3}}}{\sqrt{\left(1-R_{xy_{2}}^{2}\right)\left(1-R_{y_{2}y_{3}}^{2}\right)}}$$

Where $R_{xy_{1}y_{2}}$ is the partial correlation coefficient between variables *y_1_* and *y_2_* for fixing variable *y_3_*; $R_{xy_{1}}$ is the correlation coefficient between variables x and *y_1_*; $R_{y_{2}y_{3}}$ is the correlation coefficient between variables *y_2_* and *y_3_*; $R_{xy_{2}}$ is the correlation coefficient between variables *x* and *y_2_*. The significance test of the partial correlation coefficient is tested by student-*t*.


(4) $$t=\;\frac{R_{xy_{1}y_{2}\ldots y_{m}}}{1-R_{xy_{1}y_{2}\ldots y_{m}}^{2}}\sqrt{n-m-1}$$

Where $R_{xy_{1}y_{2}\ldots y_{m}}$ is the high-order partial correlation coefficient; *n* is the number of variables; *m* is the number of independent variables. The t distribution table can be used to determine the crucial value *t_α_*. If this is lower than *t_α_*, the partial correlation coefficient is significant, or it is nonsignificant.

## 4 Results

### 4.1 Spatiotemporal change of LAI and climate factors in the wetland of HARB

**Figure 3:**
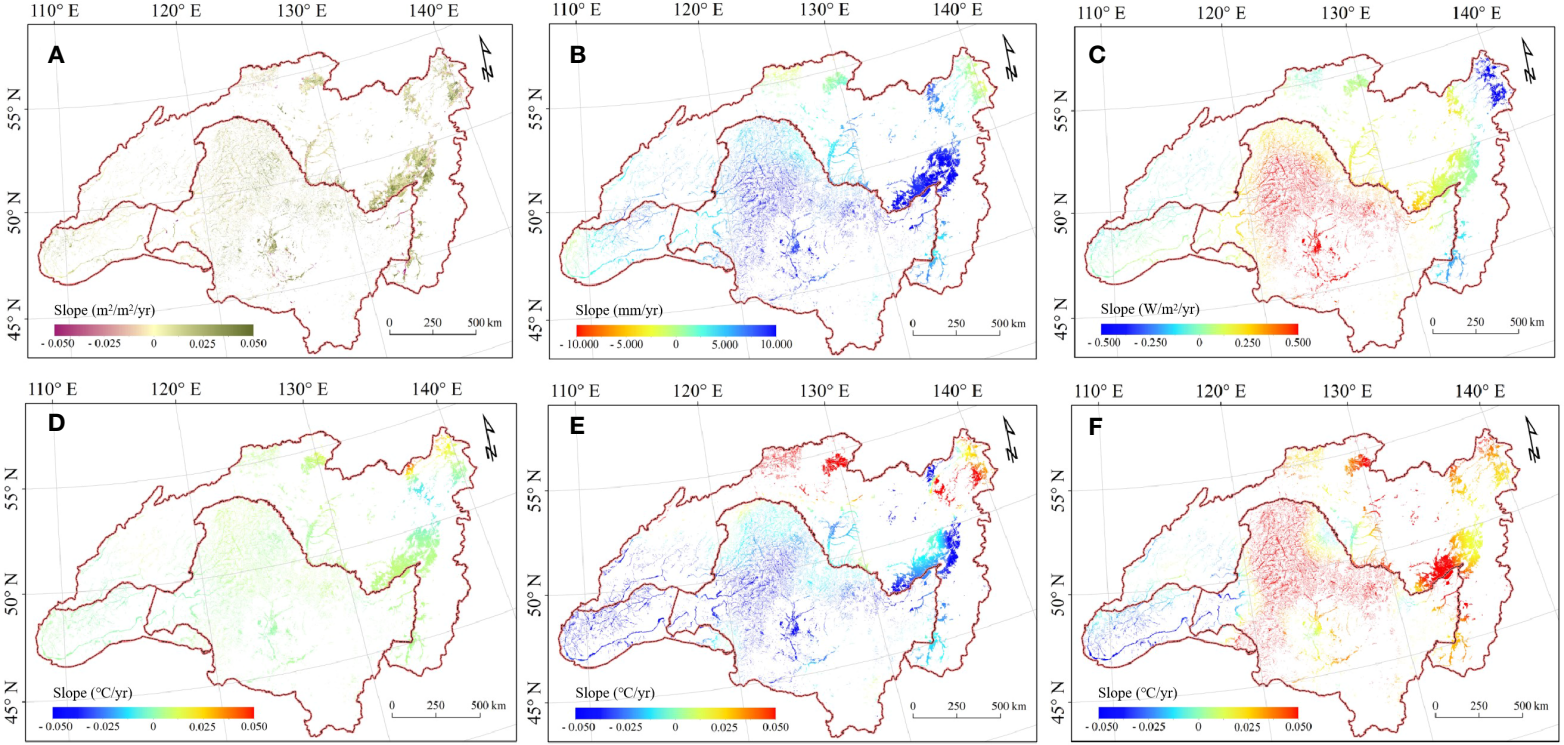
Map of trends in growing season mean LAI and climate factors between 2000-2018. **(A)** LAI. **(B)** Precipitation. **(C)** Solar radiation. **(D)** Mean temperature. **(E)** Maximum temperature. **(F)** Minimum temperature.

The illustration of the trend shows that wetland vegetation was greening in more of these pixels than it was browning (**Figure 3A**), with greening and browning defined as wetland LAI increases and decreases, respectively. The largest greening was observed in Greater Khingan, inner Mongolia, and scattered areas around the Amur River. Overall, the LAI value during the growing season has significantly increased from 2000 to 2018 (0.014 m^2^·m^-2^/yr, p = 0.012), with China having the highest growth (0.016 m^2^·m^-2^/yr, p = 0.014) followed by Russia (0.014 m^2^·m^-2^/yr, p = 0.036) and Mongolia (0.011 m^2^·m^-2^/yr, p = 0.011). Generally, the LAI increasing mainly concentrated in 0 - 0.020 m^2^/m^2^ per year (**Figure 2C**). However, there was high regional heterogeneity in the HARB. China and Russia had a valley LAI of 1.574 m^2^·m^-2^ and 1.613 m^2^·m^-2^ respectively, both occurring in 2003, while Mongolia had a lower LAI of 0.685 m^2^·m^-2^ due to its arid condition.

We further investigated the trends in climate factors including precipitation, air temperature including T_avg_, T_min_, and T_max_, and SR during the growing season across HARB (**Figures 3B–F**). For precipitation trend, the wetting zone is mostly concentrated along the Amur River, Greater and Lesser Khingan Mountains, and Stanovoy, but drying in the western Mongolia and northern Russia. The T_avg_ showed trend almost the exact opposite of precipitation in spatial distribution, and it decreased at higher elevations and latitudes. Increased air temperature were concentrated in the Songnen Plain, Heilongjiang and the Amur River, while decreased air temperatures were primarily in the Mongolian Plateau, Greater Khingan Mountain, and the highest latitude region in Russia, showing contrasting air temperature patterns in the HARB during the growing season. T_max_ demonstrated a slight upward with the increase of latitude, whereas T_min_ showed an obvious upward in Greater and Lesser Khingan mountain but decreased in Mongolia and inner Mongolia. For trend in SR, it showed an obvious downward with the increase of latitude, most prevalent in China, with higher SR trend concentrated in Songnen Plain, Mongolia and inner Mongolia, and the lower SR trend were along the Heilongjiang River and northern Russia region.

An analysis of climate data from 2000 to 2018 revealed significant upward trends in precipitation for HARB, while changes in T_avg_, T_min_, SR, and T_max_ were mostly nonsignificant (**Table 1**). Precipitation exhibited significant upward trends in China and Russia but more uniform increase in Mongolia (3.954 mm/yr, p = 0.173). T_avg_ showed nonsignificant warming trends in all three countries, while T_min_ had significant warming in China (0.050 °C/yr, p = 0. 005) and nonsignificant trends elsewhere. SR and T_max_ had nonsignificant downward trends in all three countries. These findings suggest that the HARB region was experiencing significant changes in precipitation, with some variation among countries, while other climate variables exhibit nonsignificant trends.

**Table 1:** Growing-season LAI and climate factors in HARB wetland over the period 2000-2018.

Region	HARB			China			Mongolia			Russia		
	Tend	R^2^	P	Tend	R^2^	P	Tend	R^2^	P	Tend	R^2^	P
LAI (m^2^·m^-2^/yr)	**0.014**	0.315	0.012	**0.016**	0.309	0.014	**0.011**	0.326	0.011	**0.014**	0.233	0.036
Precipitation (mm/yr)	**6.645**	0.461	0.001	**7.683**	0.431	0.002	3.954	0.106	0.173	**5.963**	0.390	0.004
SR (W·m^-2^/yr)	-0.133	0.021	0.556	-0.400	0.145	0.108	0.049	0.003	0.815	0.058	0.003	0.835
T_avg_ (°C/yr)	0.003	0.003	0.832	0.002	0.000	0.935	0.014	0.041	0.409	0.012	-0.033	0.526
T_max_ (°C/yr)	-0.021	0.030	0.487	-0.036	0.095	0.200	-0.057	0.096	0.196	-0.003	0.000	0.949
T_min_ (°C/yr)	0.033	0.177	0.073	**0.050**	0.375	0.005	-0.029	0.053	0.345	0.028	0.104	0.180

Bold shows significant correlation results.

### 4.2 Responses of vegetation dynamics to climatic change

**Figure 4:**
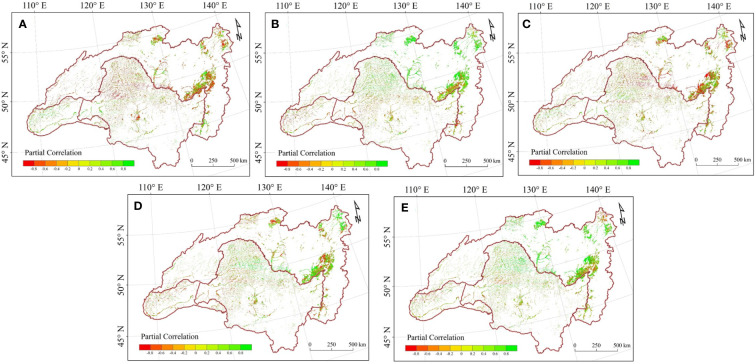
Spatial distribution of partial correlation between growing season LAI and **(A)** precipitation; **(B)** SR; **(C)** T_avg_; **(D)** T_max_; **(E)** T_min_.

To elucidate the relationships between LAI and climate change during the growing season in HARB from 2000 to 2018, we calculated partial correlations at both basin and pixel scales (**Table 2** and **Figure 4**). At the basin scale, LAI shows a nonsignificant negative correlation with precipitation, T_avg_, and T_max_ (**Figures 4A–D**); and shows a significant correlation with SR and T_min_ (**Figures 4B–E**). Concerning the pixel level, more than half of the region showed the negative connection between wetland LAI and precipitation, accounting for 59.119% of the total region, with 6.018% of which were significant at p < 0.05. While eastern Mongolia showed a positive connection with precipitation, which contributed to 60.250% positive and 12.171% significant positive of the Mongolia. In relation to SR effects, there were more pixels exhibiting a positive than a negative correlation between LAI and SR for the growing season in the HARB from 2000 to 2018, with 65.179% of which were positive and 6.767% were significant at p < 0.05, covered, almost the whole Russia region and the Greater and Lesser Khingan. Conversely, 61.111% of the negative correlations between LAl and SR concentrated in Mongolia and Inner Mongolia in China. There were more pixels exhibiting a negative than a positive correlation between LAI and T_avg_, mostly in the south of Mongolia and Inner Mongolia in China accounting for 56.056% of the whole basin. Concerning T_max_ effects, there are more pixels exhibiting a positive instead of negative correlation between LAI and T_max_, which accounted 54.527% of the whole basin, mostly located in Mongolia and Inner Mongolia in China, with 66.586% of the whole basin. Much more pixels exhibit a positive than a negative correlation between LAI and T_min_, with a positive correlation shows at the Northern of the basin, and the negative connection in Mongolia and Inner Mongolia of China.

**Table 2:** Correlation coefficients between growing season LAI and growing season climate factors for wetlands in HARB during 2000–2018.

Partial correlation	Growing season precipitation	Growing season SR	Growing season T_avg_	Growing season T_max_	Growing season T_min_
LAI of HARB wetland	-0.408	0.798^**^	-0.476	-0.166	0.689^**^
LAI of China basin wetland	-0.194	0.575^*^	-0.299	0.094	0.549^*^
LAI of Mongolia basin wetland	0.643^*^	-0.397	0.645^**^	-0.204	-0.304
LAI of Russia basin wetland	-0.156	0.465*	-0.204	-0.291	0.509

Double and single asterisks denote statistical significance at the 99% and 95% confidence levels, respectively.

For the three countries, China exhibited positive correlations between LAI and SR (0.575, p < 0.05) as well as T_min_ (0.549, p < 0.05), while precipitation and T_avg_ had nonsignificant negative correlations with LAI (-0.194 and -0.299). In Russia, LAI showed negative correlations with precipitation and T_avg_ (-0.156 and -0.204), while the correlations with SR, T_max_, and T_min_ were weak. Mongolia demonstrated strong positive correlations between LAI and both precipitation (0.643, p < 0.05) and T_avg_ (0.645, p < 0.01), with weak correlations observed for SR, T_max_, and T_min_. The highest ratio of significant positive partial correlation with LAI was found for SR in all three countries. However, Russia showed a significant negative partial correlation between LAI and T_min_ (28.473%, p < 0.05), while China and Mongolia showed the highest ratios of significant negative partial correlation between LAI and SR (41.468% and 61.112%, respectively).

### 4.3 Relationships between LAI and climate factors across different climate zones

**Figure 5:**
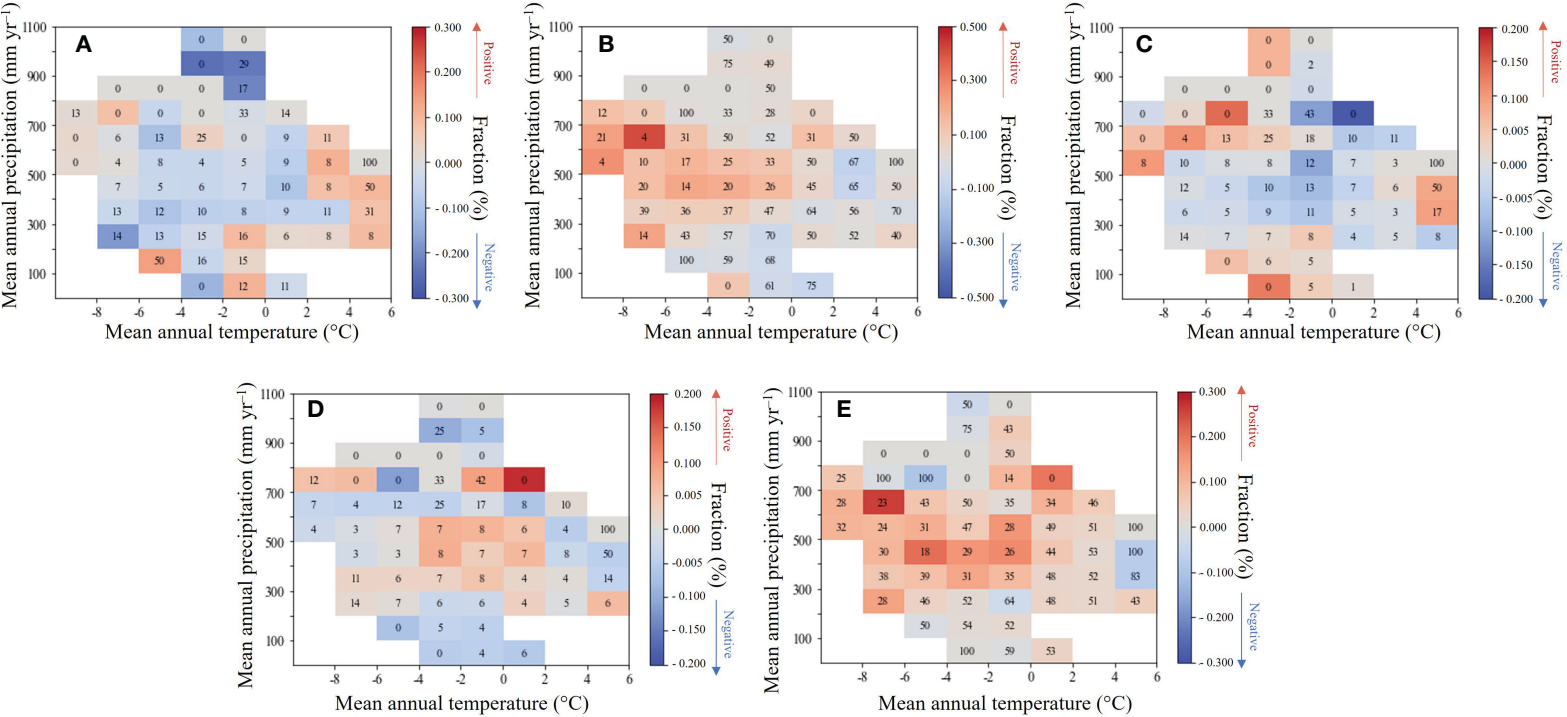
Partial correlation between growing season mean LAI and **(A)** precipitation; **(B)** SR; **(C)** T_avg_; **(D)** T_max_; **(E)** T_min_ in each 5°C interval of mean annual air temperature and 100 mm interval of mean annual precipitation climate space.

To further elaborate on the spatial diversity of the relationship between LAl and climatic factors, this research calculated the mean correlation coefficients for each 100 mm annual precipitation zone and each 2°C annual T_avg_ zone during the growing season (**Figure 5**). The right color bars indicate the mean value of spaces with the partial correlation between mean growing season LAI and climate factors, and the numbers in each interval climate space indicate the percentage of pixels with significant partial correlation (for example, if the percentage of pixels with positive partial correlation, the space is red and the number is the percentage of pixels with positive and significant partial correlation, but if the percentage of pixels with negative partial correlation, the space is blue and the number is the percentage of pixels with significant negative partial correlation).

We find that when the effects of growing season SR, T_avg_, T_max_, and T_min_ are removed, the partial correlation between LAI and precipitation was more often negative in regions as the annual precipitation intensifies, and there was an increase in the positive trend along the gradient of escalating annual air temperature (**Figure 5A**). This result signifies that an increase in precipitation is beneficial for the growth of wetland vegetation in heat sufficient areas, and when precipitation exceeds a certain threshold, it will hinder the growth of wetland vegetation. In Mongolia and Inner Mongolia in China, rainfall and wetland vegetation have a positive correlation, in contrast, Russia and northern China generally showed a negative correlation. This was also supported by the spatial correlation analyses, a stronger positive association of wetland vegetation with precipitation occurred in water limited area.

When the other climate factors are removed, SR and T_min_ delineate a similar correlation with HARB wetland LAI (**Figures 5B–E**). With the annual air temperature experiencing a reduction, the partial correlation between the two climate factors and the LAI was more often significantly positive, and the SR impact was stronger. However, there was no apparent change in the correlation along the gradient of increasing precipitation. Latitude ascendance is usually accompanied by a reduction of heat and in the higher latitude area (northern China and Russia), SR or T_min_ positive partial correlation could be found indeed. The spatial correlation results also corroborated this, showing that wetland vegetation was more strongly (and favorably) correlated with air temperature in cold regions.

There was an opposite result between the growing season T_avg_ and T_max_ (**Figures 5C, D**). As the annual precipitation and air temperature experienced an increase, the partial correlation between T_avg_ and LAI was more often negative, whilst it between T_max_ and LAI was more often positive. In a relatively warm zone, there was a substantial positive association between LAI and T_max_ when annual precipitation increased, suggesting that as the climate becomes more humid and warmer, high daytime air temperatures would promote the growth of wetland vegetation. When northern China and northeast Russia was in a warm and humid status, powerful photosynthesis promoted the wetland vegetation greening. However, in a relatively cold zone, a substantial positive association between LAI and T_avg_ was common, suggesting that in a cold and humid climate, a high T_avg_ could guarantee the normal physiological activity of wetland vegetation. Therefore, as the interannual precipitation increases in a warmer local environment, abundant rainfall and heat mean stronger photosynthesis, then accelerate the wetland vegetation growth.

## 5 Discussions

### 5.1 Climate change impacts on the HARB wetland vegetation

The study found that precipitation has a strong correlation with wetland vegetation when rainfall is insufficient to meet the demands of the Heilongjiang-Aumr River Basin ecosystem, while in air temperature-limited zones, the vegetation directly responds to air temperature changes. Past research has demonstrated that precipitation and air temperature regulate the growing season vegetation dynamics in drought and cold regions (Nemani et al., 2003; Xiao and Moody, 2004). But for the whole HARB, LAI had a nonsignificant correlation with precipitation and T_avg_ during the growing season, this may be explained by the latitudinal variation of SR, and the energy budgets of each piece of land vary according to the level of plant colonization (Anderson et al., 2003). In general, the LAI correlation with SR experienced a transition from south to north (**Figure 4B**). This trend suggested that in the mid-high region, wetland vegetation is not only dominated by air temperature but also by SR during the growing season (Nemani et al., 2003; Piao et al., 2014).

**Figure 6:**
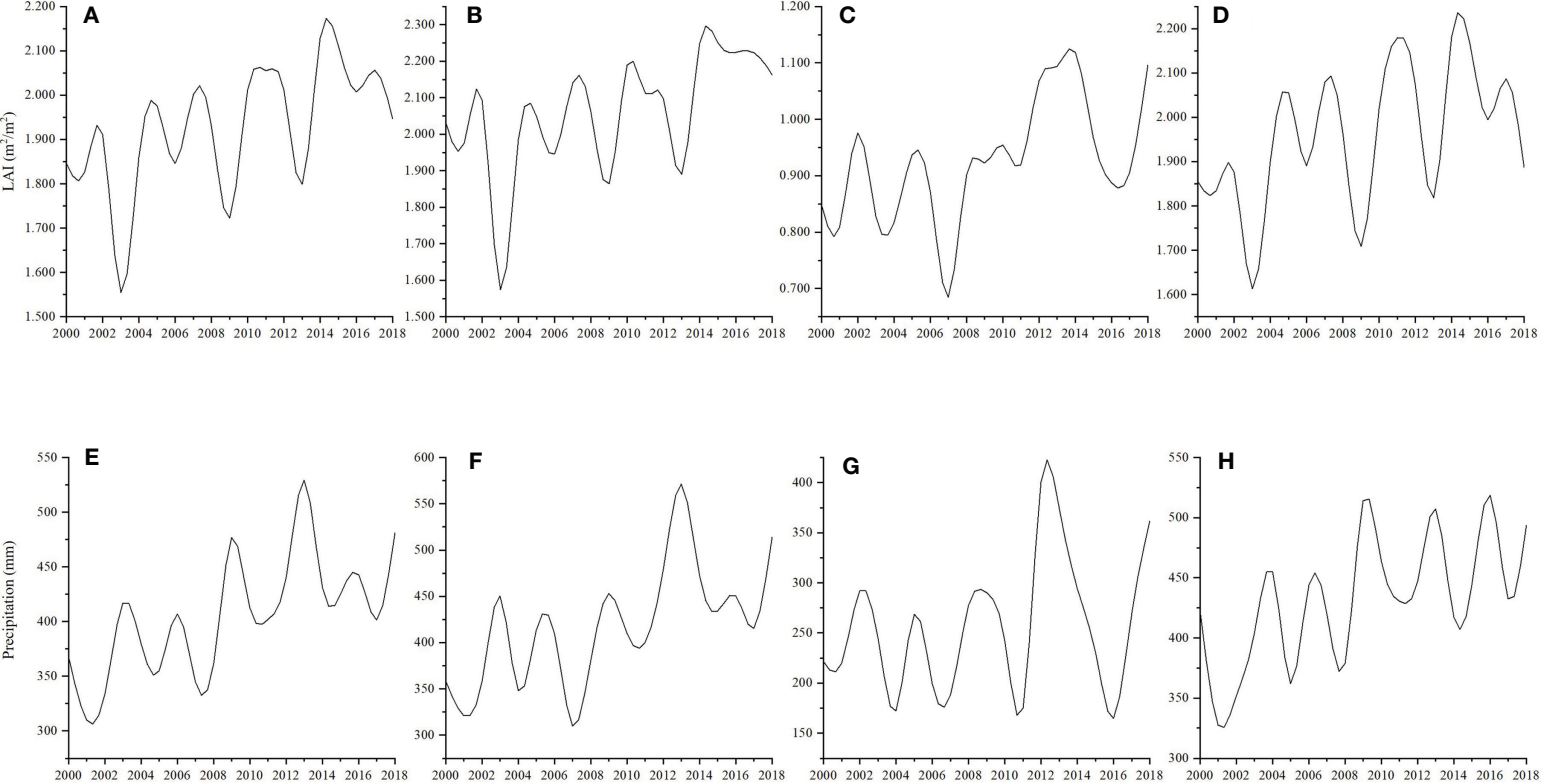
Changes in growing season LAI [**(A–D)** for HARB, China, Mongolia, and Russia, respectively] and precipitation [**(E–H) **for HARB, China, Mongolia, and Russia, respectively] from 2000 to 2018.

When the periodicity of wetland vegetation LAI was compared to the precipitation from 2000 to 2018, we found that the changes in precipitation and LAl are not synchronized. As wetland vegetation greening reflects the balance of carbon influx via photosynthesis and efflux via respiration, the time lag may indicate a more complicated greening process (Wu et al., 2015) (**Figure 6**). From 2000 to 2002, a slight time-lag effect was detected in China and Russia. This was due to the geographical location of the HARB, ecosystems with a growth period of 2 - 6 months could not respond immediately to interannual variability of climatic conditions, as well as physical limitations such as soil moisture, air temperature, and nutrient availability also taking time to change in response (Zheng et al., 2020; Yao et al., 2023). By contrast, the magnitude of Mongolia’s wetland growing season LAI was thoroughly consistent with precipitation changes. The water-limited ecosystem is more sensitive to air temperature, that is to say, in the Mongolia region of HARB, wetland vegetation could respond to hydrothermal conditions rapidly (D'Arrigo et al., 2004; Tong et al., 2017).

Conforms to the previous report, the HARB wetland in the growing season showed more quickly warms in T_min_ than T_max_ during 2000 – 2018 (Change, 2013). Vegetation growth contains two elements, photosynthesis and respiration, which are influenced by air temperature (Peng et al., 2013; Lin et al., 2020). Because photosynthesis happens during the diurnal time and the wetland vegetation in HARB is more sensitive to the minimum air temperature, whereas plant respiration occurs during both diurnal and nocturnal time and is, therefore, effect by T_max_ and T_min_ air temperature simultaneously (Wan et al., 2009). But plant respiration typically was more active in darkness, thus, T_min_ can be a more precise elucidation for respiration than T_max_ (Atkin et al., 2013). In China and Russia, where the status was wet and cold, night-time air temperature via autotrophic respiration enhancement stimulated stronger photosynthesis the following day (Belsky, 1986; Poiani and Johnson, 1993; Kim et al., 2012; Peng et al., 2013). In Mongolia, when regional moisture cannot meet the requirements of wetland vegetation growth, continuous T_avg_ rise would lead to the closure of stomata, then inhibiting vegetation growth. In the mid-high latitude area, greening will be pretending locally via attenuating SR in the arid and semi-arid areas (Mongolia and inner Mongolia), whereas the cold and moist area (China and Russia) could exacerbate warming via decreasing albedo. The positive partial correlation value in high latitude regions also implies that SR, as well as air temperature, were what limit wetland vegetation greening (Nemani et al., 2003).

### 5.2 International collaboration shapes climate change impact on HARB wetland vegetation

International management cooperation between the basin countries also shapes the anthropogenic factors of climate change in HARB wetland vegetation. As an old industrial base, the North-East of China showed slow economic growth because of the prevalence of heavy industry, state-owned enterprises, and large-scale agriculture, leading to excessive dependence on government investments (Simonov and Egidarev, 2018). In 2016, a Renewed Plan to Revitalize the Old Industrial Bases heralds a pivotal shift in focus, as the state pivots away from the extensive extraction of natural resources and towards the prioritization of high-tech manufacturing and state-owned enterprises. By shifting away from traditional sectors, the plan aims to foster sustainable economic growth and protect the natural environment in the region. The conservation of wetlands and biodiversity along transboundary rivers and lakes has been given great importance in the thirteenth Five-Year Plan for North-East China, this emphasis could potentially assist in the development of environmental water requirements for managing transboundary waters (Kenderdine, 2017). However, trade and international economic cooperation between Mongolia and Russia have been almost solely based on natural resource extraction. Furthermore, poor environmental oversight of wetland conservation and prevention, made it urgent to look for alternative, environmentally friendly development methods (Simonov and Dahmer, 2008). Only Song-Nen Plain, one of the ten major wetland habitats in HARB, is contained inside a single nation; the other nine require bilateral or trilateral collaboration to secure long-term conservation (Egidarev et al., 2016a).

Wetland vegetation of the HARB provides nature’s contribution to people which is important for all three countries. With the greening increases, the enlarged wetland leaf area enhanced evaporation and cools the region. In a moisture-limited zone, evaporation enhancement may break the water balance (Ge et al., 2020). By 2012, after the “Belt and Road” was proposed, Mongolia put forward a “Steppe Road”, as a role in the shortest transportation corridor from eastern China to western Russia (Simonov and Egidarev, 2018). Since the water scarcity in Mongolia and the poor share of wetland bands, bilateral environmental management relationships are much less jutting political status than those between China (Egidarev et al., 2016b). Because of the higher latitude and low human activities, the wetland vegetation change in Russia mostly relies on climate change, as East Asian monsoons pronounced summer floods are typical for the Far Eastern regime (Poiani and Johnson, 1993; Egidarev et al., 2016a). After the largest flood of the last century took place in 2013, the Russian government instructed the related department to hold consultations with China, including building hydraulic facilities on the Amur and its tributaries (Semenov et al., 2017). Thus, wetlands especially floodplain wetlands protection and management became scientific.

## 6 Conclusions

This study applied partial correlation analysis to draw the impacts of climatic factors (precipitation, SR, T_avg_, T_max_, and T_min_) on wetland vegetation across HARB in the growing season from 2000-2018. Generally, the growing season LAI demonstrated an upward trend, which is 0.014 m^2^·m^-2^/yr. SR and the T_min_ had a strong and significant positive influence on HARB wetland vegetation greening, which is typical in mid-high latitudes. On a regional scale, wetland vegetation in China and Russia were positively influenced by solar radiation and T_min_, while the wetland vegetation in Mongolia was positively influenced by precipitation. These correlations were further investigated at different climate intervals. We found the precipitation is positively correlated with LAI in the warm regions while is negatively correlated with LAI in the wet regions, indicating an increase in precipitation is beneficial for the growth of wetland vegetation in heat sufficient areas, and when precipitation exceeds a certain threshold, it will hinder the growth of wetland vegetation. In the cold regions, we found SR and T_min_ are positively correlated with LAI, suggesting SR and T_min_ instead of T_avg_ and T_max_ play more important roles in affecting the wetland vegetation growth in the heat limited areas. The LAI was found to be negatively correlated with T_max_ in the arid areas, indicating excessive temperature would inhibit the wetland vegetation growth when the water is limited.

To improve on the results of this paper, the wetland vegetation type in HARB could be more specific, then could trigger different wetland vegetation types in different greening diversity under the similar climate background, for instance, wetland vegetation investigation should categorize natural and constructed wetland and discuss their impact of climate change respectively. Furthermore, climate factors are far more than that, the conclusions in this paper was discussed by using the 5 commenly used variables. In the follow-up studies, we could concentrate on these two aspects when conducting the study.

## Data availability statement

Publicly available datasets were analyzed in this study. This data can be found here: https://www.ncei.noaa.gov/maps-and-geospatial-products, http://data.cma.cnUSGS, http://glovis.usgs.gov/https://www.nature.com/articles/sdata2017191.

## Author contributions

XC: methodology, software, writing - original draft, writing - review & editing, formal analysis, validation. LY: conceptualization, methodology, funding acquisition, formal analysis, software, writing - review & editing. GL: software, resources, investigation, writing - review & editing. XL and LB: data curation, software. All authors contributed to the article and approved the submitted version.

## References

[B1] Abatzoglou J. T. Dobrowski S. Z. Parks S. A. Hegewisch K. C. (2018). TerraClimate, a high-resolution global dataset of monthly climate and climatic water balance from 1958–2015. Sci. Data 5 (1), 1–12. doi: 10.1038/sdata.2017.191 29313841 PMC5759372

[B2] Anderson F. E. Snyder R. L. Miller R. L. Drexler J. (2003). A micrometeorological investigation of a restored California wetland ecosystem. Bull. Am. Meteorological Soc. 84 (9), 1170–1172. doi: 10.1175/BAMS-84-9-1170

[B3] Atkin O. K. Turnbull M. H. Zaragoza-Castells J. Fyllas N. M. Lloyd J. Meir P.. (2013). Light inhibition of leaf respiration as soil fertility declines along a post-glacial chronosequence in new Zealand: an analysis using the kok method. Plant Soil 367 (1), 163–182. doi: 10.1007/s11104-013-1686-0

[B5] Avis C. A. Weaver A. J. Meissner K. J. (2011). Reduction in areal extent of high-latitude wetlands in response to permafrost thaw. Nat. Geosci. 4 (7), 444–448. doi: 10.1038/ngeo1160

[B6] Bao G. Qin Z. Bao Y. Zhou Y. Li W. Sanjjav A. (2014). NDVI-based long-term vegetation dynamics and its response to climatic change in the Mongolian plateau. Remote Sens. 6 (9), 8337–8358. doi: 10.3390/rs6098337

[B7] Belsky A. J. (1986). Does herbivory benefit plants? a review of the evidence. Am. Nat. 127 (6), 870–892. doi: 10.1086/284531

[B8] Burkett V. Kusler J. (2000). Climate change: potential impacts and interactions IN wetlands OF the untted states 1. JAWRA J. Am. Water Resour. Assoc. 36 (2), 313–320. doi: 10.1111/j.1752-1688.2000.tb04270.x

[B9] Chang Z. Gong H. Zhang J. Chen M. (2014). Correlation analysis on interferometric coherence degree and probability of residue occurrence in interferogram. IEEE Sensors J. 14 (7), 2369–2375. doi: 10.1109/JSEN.2014.2310751

[B10] Change I. C. (2013). The physical science basis. Contribution working group I to fifth Assess. Rep. intergovernmental panel Climate Change 1535 2013.

[B11] Chen J. M. Black T. (1992). Defining leaf area index for non-flat leaves. Plant Cell Environ. 15 (4), 421–429. doi: 10.1111/j.1365-3040.1992.tb00992.x

[B12] Chen I.-C. Hill J. K. Ohlemüller R. Roy D. B. Thomas C. D. (2011). Rapid range shifts of species associated with high levels of climate warming. Science 333 (6045), 1024–1026. 21852500 10.1126/science.1206432

[B13] Chen T. Tang G. Yuan Y. Guo H. Xu Z. Jiang G. (2020). Unraveling the relative impacts of climate change and human activities on grassland productivity in Central Asia over last three decades. Sci. of The Total Environ. 743, 14064. doi: 10.1016/j.scitotenv.2020.140649 32758823

[B14] Chu H. Venevsky S. Wu C. Wang M. (2019). NDVI-based vegetation dynamics and its response to climate changes at amur-heilongjiang river basin from 1982 to 2015. Sci. Total Environ. 650, 2051–2062. doi: 10.1016/j.scitotenv.2018.09.115 30290347

[B15] Chuai X. Huang X. Wang W. Bao G. (2013). NDVI, temperature and precipitation changes and their relationships with different vegetation types during 1998–2007 in inner Mongolia, China. Int. J. Climatol. 33 (7), 1696–1706. doi: 10.1002/joc.3543

[B16] D'Arrigo R. D. Kaufmann R. K. Davi N. Jacoby G. C. Laskowski C. Myneni R. B.. (2004). Thresholds for warming-induced growth decline at elevational tree line in the Yukon territory, Canada. Global Biogeochemical Cycles 18 (3). doi: 10.1029/2004GB002249

[B17] De Jong R. de Bruin S. de Wit A. Schaepman M. E. Dent D. L. (2011). Analysis of monotonic greening and browning trends from global NDVI time-series. Remote Sens. Environ. 115 (2), 692–702. doi: 10.1016/j.rse.2010.10.011

[B18] Díaz S. M. Settele J. Brondízio E. Ngo H. Guèze M. Agard J.. (2019). The global assessment report on biodiversity and ecosystem services: summary for policy makers.

[B19] Du J. He P. Fang S. Liu W. Yuan X. Yin J. (2017). Autumn NDVI contributes more and more to vegetation improvement in the growing season across the Tibetan plateau. Int. J. Digital Earth 10 (11), 1098–1117. doi: 10.1007/978-94-007-4001-3_7/10.1080/17538947.2017.1282547

[B20] Egidarev E. Simonov E. Darman Y. (2016a). “Amur-heilong river basin: overview of wetland resources,” in The wetland book: II: distribution, description and conservation. Eds. Finlayson C. M. Milton G. R. Prentice R. C. Davidson N. C. (Dordrecht: Springer Netherlands), 1–15.

[B21] Egidarev E. Simonov E. Darman Y. (2016b). Amur-heilong river basin: overview of wetland resources. Wetland Book 2, 1–15.

[B22] French N. H. Kasischke E. S. Hall R. J. Murphy K. A. Verbyla D. L. Hoy E. E.. (2008). Using landsat data to assess fire and burn severity in the north American boreal forest region: an overview and summary of results. Int. J. Wildland Fire 17 (4), 443–462. doi: 10.1007/978-94-007-4001-3_7/10.1071/wf08007

[B23] Gallego-Sala A. V. Charman D. J. Brewer S. Page S. E. Prentice I. C. Friedlingstein P.. (2018). Latitudinal limits to the predicted increase of the peatland carbon sink with warming. Nat. Climate Change 8 (10), 907–913. doi: 10.1038/s41558-018-0271-1

[B24] Ge J. Pitman A. J. Guo W. Zan B. Fu C. (2020). Impact of revegetation of the loess plateau of China on the regional growing season water balance. Hydrology Earth System Sci. 24 (2), 515–533. doi: 10.5194/hess-24-515-2020

[B25] Goetz S. J. Bunn A. G. Fiske G. J. Houghton R. A. (2005). Satellite-observed photosynthetic trends across boreal north America associated with climate and fire disturbance. Proc. Natl. Acad. Sci. 102 (38), 13521–13525. doi: 10.1073/pnas.0506179102 16174745 PMC1224647

[B26] Hijmans R. J. Cameron S. E. Parra J. L. Jones P. G. Jarvis A. (2005). Very high resolution interpolated climate surfaces for global land areas. Int. J. Climatology: A J. R. Meteorological Soc. 25 (15), 1965–1978. doi: 10.1002/joc.1276

[B27] IPCC (2013). Climate change 2013: the physical science basis. Contribution working group I to fifth Assess. Rep. intergovernmental panel Climate Change 1535.

[B28] Islam A. S. Bala S. K. (2008). Assessment of potato phenological characteristics using MODIS-derived NDVI and LAI information. GIScience & Remote Sensing 45, 454–470. doi: 10.1007/978-94-007-4001-3_7/10.2747/1548-1603.45.4.454

[B29] Ji L. Fan K. (2020). Effect of atlantic sea surface temperature in May on intraseasonal variability of Eurasian NDVI in summer. J. Geophysical Res.: Atmospheres 125 (9), e2019JD031991. doi: 10.1029/2019JD031991

[B30] Jiang L. Bao A. Guo H. Ndayisaba F. (2017). Vegetation dynamics and responses to climate change and human activities in central Asia. Sci. Total Environ. 599, 967–980. doi: 10.1016/j.scitotenv.2017.05.012 28505889

[B31] Kala J. Decker M. Exbrayat J.-F. Pitman A. J. Carouge C. Evans J. P.. (2014). Influence of leaf area index prescriptions on simulations of heat, moisture, and carbon fluxes. J. Hydrometeorol. 15 (1), 489–503. doi: 10.1175/JHM-D-13-063.1

[B32] Kayranli B. Scholz M. Mustafa A. Hedmark Å. (2010). Carbon storage and fluxes within freshwater wetlands: a critical review. Wetlands 30 (1), 111–124. doi: 10.1007/s13157-009-0003-4

[B33] Kenderdine T. (2017). China's industrial policy, strategic emerging industries and space law. Asia Pacific Policy Stud. 4 (2), 325–342. doi: 10.1002/app5.177

[B34] Kim Y. Kimball J. S. Zhang K. McDonald K. C. (2012). Satellite detection of increasing northern hemisphere non-frozen seasons from 1979 to 2008: implications for regional vegetation growth. Remote Sens. Environ. 121, 472–487. doi: 10.1016/j.rse.2012.02.014

[B35] Lahmer W. Pfützner B. Becker A. (2001). Assessment of land use and climate change impacts on the mesoscale. Phys. Chem. Earth Part B: Hydrology Oceans Atmosphere 26 (7-8), 565–575. doi: 10.1016/S1464-1909(01)00051-X

[B36] Lin X. Niu J. Berndtsson R. Yu X. Zhang L. Chen X. (2020). Ndvi dynamics and its response to climate change and reforestation in northern China. Remote Sens. 12 (24) 4138. doi: 10.3390/rs12244138

[B37] Liu Y. Lei H. (2015). Responses of natural vegetation dynamics to climate drivers in China from 1982 to 2011. Remote Sens. 7 (8), 10243–10268. doi: 10.3390/rs70810243

[B38] Liu T. Yu L. Bu K. Yang J. Yan F. Zhang S.. (2022). Thermal and moisture response to land surface changes across different ecosystems over heilong-amur river basin. Sci. Total Environ. 818, 151799. doi: 10.1016/j.scitotenv.2021.151799 34801503

[B39] Lucht W. Prentice I. C. Myneni R. B. Sitch S. Friedlingstein P. Cramer W.. (2002). Climatic control of the high-latitude vegetation greening trend and pinatubo effect. Science 296 (5573), 1687–1689. 12040194 10.1126/science.1071828

[B40] Masson-Delmotte V. Zhai P. Pirani A. Connors S. L. Péan C. Berger S.. (2021). Climate change 2021: the physical science basis. Contribution working group I to sixth Assess. Rep. intergovernmental panel Climate Change 2. doi: 10.1007/978-94-007-4001-3_7

[B41] Meng M. Huang N. Wu M. Pei J. Wang J. Niu Z. (2019). Vegetation change in response to climate factors and human activities on the Mongolian plateau. PeerJ 7, e7735. doi: 10.7717/peerj.7735 31592100 PMC6776067

[B42] Menzel A. Fabian P. (1999). Growing season extended in Europe. Nature 397 (6721), 659–659. doi: 10.1007/978-94-007-4001-3_7/10.1038/17709

[B43] Menzel A. Sparks T. H. Estrella N. Koch E. Aasa A. Ahas R.. (2006). European Phenological response to climate change matches the warming pattern. Global Change Biol. 12 (10), 1969–1976. doi: 10.1111/j.1365-2486.2006.01193.x

[B44] Millennium ecosystem assessment (2005). Ecosystems and human well-being (Washington, DC: Island press).

[B45] Montandon L. M. Small E. E. (2008). The impact of soil reflectance on the quantification of the green vegetation fraction from NDVI. Remote Sens. Environ. 112 (4), 1835–1845. doi: 10.1016/j.rse.2007.09.007

[B46] Myneni R. B. Keeling C. D. Tucker C. J. Asrar G. Nemani R. R. (1997). Increased plant growth in the northern high latitudes from 1981 to 1991. Nature 386 (6626), 698–702. doi: 10.1038/386698a0

[B47] Nemani R. R. Keeling C. D. Hashimoto H. Jolly W. M. Piper S. C. Tucker C. J.. (2003). Climate-driven increases in global terrestrial net primary production from 1982 to 1999. science 300 (5625), 1560–1563. 12791990 10.1126/science.1082750

[B48] Peng S. Chen A. Xu L. Cao C. Fang J. Myneni R. B.. (2011). Recent change of vegetation growth trend in China. Environ. Res. Lett. 6 (4), 044027. doi: 10.1088/1748-9326/6/4/044027

[B49] Peng S. Piao S. Ciais P. Myneni R. B. Chen A. Chevallier F.. (2013). Asymmetric effects of daytime and night-time warming on northern hemisphere vegetation. Nature 501 (7465), 88–92. doi: 10.1038/nature12434 24005415

[B50] Peng S.-S. Piao S. Zeng Z. Ciais P. Zhou L. Li L. Z.. (2014). Afforestation in China cools local land surface temperature. Proc. Natl. Acad. Sci. 111 (8), 2915–2919. doi: 10.1073/pnas.1315126111 24516135 PMC3939881

[B51] Pervushina N. (2012). Water management and use in the amur-heilong river basin: challenges and prospects. Environ. Secur. watersheds: sea Azov, 223–240. doi: 10.1007/978-94-007-2460-0_13

[B52] Piao S. Cui M. Chen A. Wang X. Ciais P. Liu J.. (2011). Altitude and temperature dependence of change in the spring vegetation green-up date from 1982 to 2006 in the qinghai-xizang plateau. Agric. For. Meteorology 151 (12), 1599–1608. doi: 10.1016/j.agrformet.2011.06.016

[B53] Piao S. Fang J. Zhou L. Guo Q. Henderson M. Ji W.. (2003). Interannual variations of monthly and seasonal normalized difference vegetation index (NDVI) in China from 1982 to 1999. J. Geophysical Res: Atmospheres 108 (D14).

[B54] Piao S. Nan H. Huntingford C. Ciais P. Friedlingstein P. Sitch S.. (2014). Evidence for a weakening relationship between interannual temperature variability and northern vegetation activity. Nat. Commun. 5 (1) 5018. doi: 10.1038/ncomms6018 25318638

[B55] Poiani K. A. Johnson W. C. (1993). Potential effects of climate change on a semi-permanent prairie wetland. Climatic Change 24 (3), 213–232. doi: 10.1007/BF01091830

[B56] Robinson N. P. Allred B. W. Smith W. K. Jones M. O. Moreno A. Erickson T. A.. (2018). Terrestrial primary production for the conterminous united states derived from landsat 30 m and MODIS 250 m. Remote Sens. Ecol. Conserv. 4 (3), 264–280. doi: 10.1002/rse2.74

[B57] Ross J. (1981). The radiation regime and architecture of plant stands (Springer Science & Business Media).

[B58] Salimi S. Almuktar S. A. Scholz M. (2021). Impact of climate change on wetland ecosystems: a critical review of experimental wetlands. J. Environ. Manage. 286, 112160. doi: 10.1016/j.jenvman.2021.112160 33611067

[B59] Semenov E. Sokolikhina N. Tatarinovich E. (2017). Monsoon circulation over the amur river basin during catastrophic flood and extreme drought in summer. Russian Meteorology Hydrology 42, 141–149. doi: 10.3103/S1068373917030013

[B60] Simonov E. A. Dahmer T. D. (2008). Amur-Heilong river basin reader. Ecosyst. Hong Kong.

[B61] Simonov E. Egidarev E. (2018). Intergovernmental cooperation on the amur river basin management in the twenty-first century. Int. J. Water Resour. Dev. 34 (5), 771–791. doi: 10.1080/07900627.2017.1344122

[B62] Smith C. (2004). Environmental physics (Routledge).

[B63] Tong S. Zhang J. Bao Y. (2017). Spatial and temporal variations of vegetation cover and the relationships with climate factors in inner Mongolia based on GIMMS NDVI3g data. J. Arid Land 9 (3), 394–407. doi: 10.1007/s40333-017-0016-4

[B64] Turner R. K. Van Den Bergh J. C. Söderqvist T. Barendregt A. van der Straaten J. Maltby E.. (2000). Ecological-economic analysis of wetlands: scientific integration for management and policy. Ecol. Economics 35 (1), 7–23. doi: 10.1016/S0921-8009(00)00164-6

[B65] Wan S. Xia J. Liu W. Niu S. (2009). Photosynthetic overcompensation under nocturnal warming enhances grassland carbon sequestration. Ecology 90 (10), 2700–2710. doi: 10.1890/08-2026.1 19886480

[B66] Wang Y. Shen X. Jiang M. Lu X. (2020). Vegetation change and its response to climate change between 2000 and 2016 in marshes of the songnen plain, northeast China. Sustainability 12 (9), 3569. doi: 10.3390/su12093569

[B67] Wei D. Zhang X. Wang X. (2017). Strengthening hydrological regulation of China's wetland greenness under a warmer climate. J. Geophysical Res.: Biogeosci. 122 (12), 3206–3217. doi: 10.1002/2017JG004114

[B68] Wu D. Zhao X. Liang S. Zhou T. Huang K. Tang B.. (2015). Time-lag effects of global vegetation responses to climate change. Global Change Biol. 21 (9), 3520–3531. doi: 10.1111/gcb.12945 25858027

[B69] Xiao J. Moody A. (2004). Trends in vegetation activity and their climatic correlates: China 1982 to 1998. Int. J. Remote Sens. 25 (24), 5669–5689. doi: 10.1080/01431160410001735094

[B70] Yan X. Wang R. Niu Z. (2022). Response of china’s wetland NDVI to climate changes. Wetlands 42 (6), 55. doi: 10.1007/978-94-007-4001-3_7/10.1007/s13157-022-01568-0

[B71] Yang R. Li X. Mao D. Wang Z. Tian Y. Dong Y. (2020). Examining fractional vegetation cover dynamics in response to climate from 1982 to 2015 in the amur river basin for SDG 13. Sustainability 12 (14), 5866. doi: 10.3390/su12145866

[B72] Yao Y. Liu Y. Zhou S. Song J. Fu B. (2023). Soil moisture determines the recovery time of ecosystems from drought. Global Change Biol. doi: 10.1111/gcb.16620 36708329

[B73] Yu L. Liu Y. Bu K. Wang W. J. Zhang S. (2022). Soil temperature mitigation due to vegetation biophysical feedbacks. Global Planetary Change 218, 103971. doi: 10.1016/j.gloplacha.2022.103971

[B74] Yu L. Liu T. Bu K. Yang J. Chang L. Zhang S. (2017). Influence of snow cover changes on surface radiation and heat balance based on the WRF model. Theor. Appl. Climatology 130, 205–215. doi: 10.1007/s00704-016-1856-0

[B75] Yu L. Liu Y. Liu T. Yan F. (2020). Impact of recent vegetation greening on temperature and precipitation over China. Agric. For. Meteorology 295, 108197. doi: 10.1016/j.agrformet.2020.108197

[B76] Zeng Z. Piao S. Li L. Z. Zhou L. Ciais P. Wang T.. (2017). Climate mitigation from vegetation biophysical feedbacks during the past three decades. Nat. Climate Change 7 (6), 432–436. doi: 10.1038/nclimate3299

[B77] Zhang X. Tang Q. Zheng J. Ge Q. (2013). Warming/cooling effects of cropland greenness changes during 1982–2006 in the north China plain. Environ. Res. Lett. 8 (2), 24038. doi: 10.1088/1748-9326/8/2/024038

[B78] Zhang G. Xu X. Zhou C. Zhang H. Ouyang H. (2011). Responses of grassland vegetation to climatic variations on different temporal scales in hulun buir grassland in the past 30 years. J. Geographical Sci. 21 (4), 634–650. doi: 10.1007/s11442-011-0869-y

[B79] Zhang Z. Yao Q. Liu K.-b. Li L. Yin R. Wang G.. (2021). Historical flooding regime along the amur river and its links to East Asia summer monsoon circulation. Geomorphology 388, 107782. doi: 10.1016/j.geomorph.2021.107782

[B80] Zheng J. Xu X. Jia G. Wu W. (2020). Understanding the spring phenology of Arctic tundra using multiple satellite data products and ground observations. Sci. China Earth Sci. 63, 1599–1612. doi: 10.1007/s11430-019-9644-8

